# The kinase domain of PFKFB4 is required to stimulate the glucose metabolism and growth of H460 xenografts

**DOI:** 10.1186/2049-3002-2-S1-P74

**Published:** 2014-05-28

**Authors:** J Clark, Y Imbert-Fernandez, J Chesney, S Telang

**Affiliations:** 1 University of Louisville, Louisville, KY, USA

## Background

Oncogenic activation and HIF-1 overexpression stimulate glycolysis in part by activating the bifunctional 6-phosphofructo-2-kinase/fructose-2,6-bisphosphatases (PFKFB1-4) that synthesize fructose-2,6-bisphosphate (F2,6BP), an allosteric activator of 6-phosphofructo-1-kinase (PFK-1), which is an essential control point in the glycolytic pathway. Although PFKFB3 is considered the dominant source of F2,6BP due to its high kinase activity and expression in multiple tumor types, the activity and function of the other PFKFBs has not been fully explored. Interestingly, the PFKFB4 family member was recently found in two independent and unbiased RNAi-based screens to be required for cancer cell survival. We sought to examine the specific requirements of PFKFB4 in regulating glycolytic flux relative to the pentose shunt, two pathways required for cell survival.

## Methods

Recombinant human PFKFB4 was synthesized and examined for activity. PFKFB4 protein expression was measured by Western blotting after inhibition with siRNA. For NMR, cells were grown in 13C-labeled glucose and spectra captured for analysis. Xenograft experiments were conducted.

## Results

We first sought to examine the kinase and phosphatase activities of human recombinant PFKFB4, which have not previously been determined. We expressed and purified human PFKFB4 and found that the enzyme was bifunctional: Km (fructose-6-phosphate, F6P) = 374.2± 20 μM; Km (F2,6BP) = 43.52± 5 μM; kinase:phosphatase ratio = 4.6:1. We then silenced PFKFB4 using siRNA in 4 human cancer cell lines (H460, A549, LNCaP and HCT116, scrambled siRNA as control) and found that PFKFB4 knockdown significantly decreased the steady-state [F2,6BP], [ATP] and ^13^C-glycolytic flux to lactate and glutamate. Importantly, the requirement of PFKFB4 for ^13^C-glycolytic flux to lactate was significantly enhanced under hypoxic conditions. We did not observe any effects of PFKFB4 inhibition on flux through the oxidative pentose shunt as measured by the conversion of ^13^C-glucose into purine and pyrimidine riboses and by steady-state concentration of NAPDH. Last, we found that PFKFB4 inhibition suppressed H460 xenograft [F2,6BP], glucose uptake and growth in athymic mice.

## Conclusions

These data indicate that PFKFB4 has greater kinase than phosphatase activity and predominantly functions to synthesize F2,6BP and activate glycolytic flux into the 3-carbon portion of the glycolytic pathway. This is not surprising given that the concentration of the substrate for the kinase reaction (F6P) is several thousand-fold higher than the substrate for the phosphatase reaction (F2,6BP) in normal and transformed cells. Lastly, we believe that the increased requirement of PFKFB4 for the regulation of glycolysis in response to hypoxia indicates that small molecule inhibitors of PFKFB4 may have utility as anti-neoplastic agents.

